# Ultrasound identification of the cementoenamel junction and clinical correlation through ex vivo analysis

**DOI:** 10.1038/s41598-024-79081-z

**Published:** 2024-11-13

**Authors:** Baiyan Qi, Lei Fu, Tamer Abdelrehim, Jason J. Chang, Harrison Chang, Casey Chen, Jesse V. Jokerst

**Affiliations:** 1 Aiiso Yufeng Li Family Department of Chemical and Nano Engineering, University of California, San Diego, La Jolla, CA 92093 USA; 2 Herman Ostrow School of Dentistry, University of Southern California, 925 West 34th Street, Los Angeles, CA USA; 3 Material Science and Engineering Program, University of California, San Diego, La Jolla, CA 92093 USA; 4 Radiology Department, University of California, San Diego, La Jolla, CA 92093 USA

**Keywords:** Biological techniques, Biotechnology, Anatomy, Biomarkers, Health care

## Abstract

Accurately identifying periodontal landmarks via acoustic imaging is increasingly important. Here, we evaluated the accuracy of cementoenamel junction (CEJ) identification using ultrasound by comparing it to clinical methods in 153 extracted human teeth. The distance between the CEJ to a reference point was measured using two clinical methods (visual examination and tactile sensation) as well as ultrasound imaging. Statistical analyses were performed across all teeth and sub-groups, including tooth types (incisors, cuspids, and molars/premolars), and two classifications: A- vs. B- (visually detectable or undetectable CEJ, respectively); and CL-S vs. CL-D (shallow or deep cervical lesions). In A- or CL-S teeth, ultrasound measurements highly agreed with clinical measurements, showing a 1.72-mm 95% CI for A- and 1.99-mm 95% CI for CL-S compared to visual examination, and a 1.77-mm 95% CI for A- and a 2.10-mm 95% CI for CL-S compared to tactile sensation, respectively. For 80% of A- and 76% of CL-S teeth, the difference between ultrasound and visual examination was within ± 20%. For 81% of A- and 80% of CL-S teeth, the difference between ultrasound and tactile sensation was within ± 20%. The variance of ultrasound versus clinical CEJ identifications showed a significant correlation (*r* = 0.6607) to the cervical lesion depth. The errors between ultrasound and clinical measurements show no significant bias across different tooth types.

**Supplementary Information:**

The online version contains supplementary material available at 10.1038/s41598-024-79081-z.

## Introduction

Periodontitis is a common inflammatory disease attributed to microbial dysbiosis and the associated host response leading to loss of tooth-supporting structures^1,2^. Periodontal diagnosis relies on clinical probing and measurement of periodontal probing depth (PPD), clinical attachment loss (CAL), and gingival recession. While probing is a routine clinical task, it is time-consuming and error-prone due to variations in probing force^3,4^, the insertion point, and the probing angulation^5,6^.

Ultrasound imaging has been increasingly studied as an alternative method for assessing peri-implant and periodontal health^7–14^. Ultrasound imaging provides information on both the hard and soft tissues of teeth and periodontium. An important landmark in periodontal diagnosis is the cementoenamel junction (CEJ), where the tooth enamel meets the root cementum. The CEJ is a reference point to determine the amount of CAL, gingival recession, and alveolar bone level. These parameters can then be used to diagnose periodontal disease or monitor treatment. Proper CEJ identification is thus a crucial step in the periodontal examination.

The CEJ can be identified visually at sites with advanced gingival recession. In the absence of gingival recession, the location of the CEJ is determined by tactile sensing using a periodontal probe subgingivally^15^. In contrast, ultrasound imaging can detect the CEJ regardless of whether it is supra- or subgingival^14,16–18^. The resolution of ultrasound imaging depends on several factors. Ultrasound with high frequency (> 20 MHz), elevational focusing via an acoustic lens, proper channel numbers (usually 256), and advanced beamforming and apodization processing can identify the CEJ and other dental landmarks with sub-100-µm resolution when images are collected with sufficient contrast^19^. The CEJ can also be detected in radiography, but ultrasound imaging has been shown to be as accurate as radiography in assessing periodontal tissues without the potential hazard from ionizing radiation. Indeed, a 2018 meta-analysis of four porcine and human cadaver studies^20–23^ compared cone-beam CT (CBCT) to ultrasound imaging to determine the alveolar bone level by measuring the distance between CEJ and alveolar bone crest (ABC). The results showed that the mean difference between CBCT and ultrasound was less than 8.8%^24^.

In a previous study, we imaged 66 teeth from 16 subjects and demonstrated that ultrasound imaging was as effective in stratifying periodontal disease severity as clinical assessment^25^. Interestingly, we noted significant inter-examiner variations in measurements that relied on CEJ as a reference point (e.g., the distance between CEJ and ABC), possibly due to difficulty locating CEJ. Ultrasound physics^26^ dictates that the CEJ should be more challenging to identify than other landmarks: The CEJ is the intersection of two hard tissues with minimal differences in acoustic impedance. A lower difference in acoustic impedance between two tissues implies lower contrast in the images, thus making the CEJ potentially harder to detect. Moreover, the CEJ can be affected by noncarious cervical lesions (NCCLs), which may hamper CEJ detection by ultrasound imaging (and visually). In contrast, the ABC or gingival margin (GM) are easily identified based on their anatomic features in ultrasound imaging.

This study aimed to compare the accuracy of CEJ detection of extracted human teeth by ultrasound imaging to visual examination or tactile sensation. Previously extracted teeth were included and grouped based on tooth types (incisors, cuspids, molars/premolars), the integrity of the CEJ by a modification of the American Academy of Periodontology classification^27^ (Class A- and Class B-), and the depth of the cervical lesions as shallow as ≤ 0.2 mm or as deep as > 0.2 mm (Class CL-S and Class CL-D). Two small ball bearings were glued to the mid-labial/mudball surface of the crown and root to provide reference points for orientation and measurements. An experienced clinician (C.C.) identified the CEJ by visual examination and tactile sensation with a periodontal probe. The distances between the top ball bearing and the CEJ were measured with a caliper. We then identified the CEJ and measured its distance to the top ball bearing using ultrasound imaging. The results reflect the value of ultrasound imaging in making oral health diagnosis that involves the CEJ.

## Methods and materials

### Preparation of extracted teeth

All work was approved by the institutional review boards of the University of Southern California. All the subjects provided their written informed consent before imaging. Teeth were extracted as part of routine dental treatment in the clinic. The teeth were stored in 0.5% sodium hypochloride. Teeth with the following characteristics were excluded from this study: extensive restorations, large fractures, large caries, root caries at the buccal/labial surface, cervical lesions of 1 mm or greater in depth, and teeth treated endodontically. Teeth with composite restorations over the CEJ were also excluded. Selected teeth (*n* = 157) were rinsed with water, dried, and sterilized by autoclaving in a standard condition (250 °F for 30 min).

Two small steel ball bearings (0.5 mm in diameter) were glued to the mid-labial or mid-buccal surface of the tooth, one above and one below the CEJ. The spacing between the two ball bearings was kept within 10 mm to ensure they were both within the 14.07 mm field of view of the transducer. The 0.5 mm diameter was chosen to balance ease of manual handling and minimal surface coverage of the tooth. These two ball bearings formed a plane that bisected the labial or buccal surface for orientation. The top ball bearing on the enamel surface provided a reference to measure the distance to the CEJ.

### Detecting CEJ by visual examination and tactile sensation

An experienced clinician (C.C.) marked the location of each tooth’s CEJ by visual examination and then by tactile sensation. First, the location of the CEJ was identified visually and marked with a dot using a black marker pen as close to the center line formed by the two reference ball bearings. For teeth with deep NCCLs, the location of the CEJ was estimated by drawing a line from the intact mesial and distal CEJ. Next, the tooth was covered with a cloth, and the CEJ was detected by tactile sensation with a #12 Marquis probe (Henry Schein, Melville, NY). The probe traversed corono-apically on the tooth surface, simulating the same detection motion for CEJ in the clinical setting^15^. A dot was placed at the CEJ with a blue marker pen close to the reference mid-line.

**Fig. 1:**
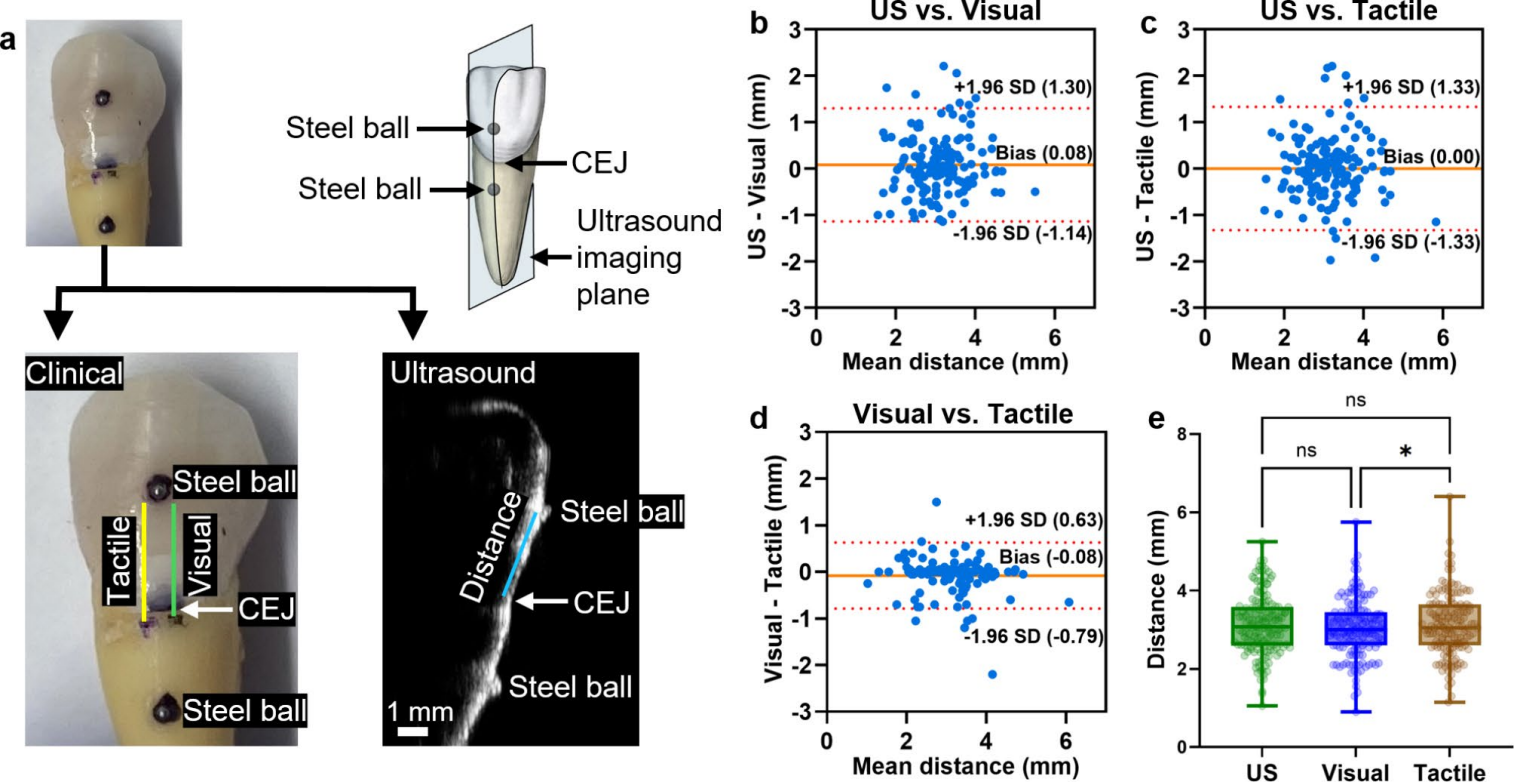
Overview of the CEJ identification using ultrasound and clinical measurements. (**a)** On each tooth, the distance between top steel ball to CEJ was measured by clinical measurements including tactile sensation and visual examination (left) and ultrasound (right). Note that the composite restoration margin was away from the CEJ and did not affect the measurements. **(b)–(d)** Bland-Altman plots of distances measured by US vs. Visual, US vs. Tactile, and Visual vs. Tactile on all teeth (*n* = 153), respectively. Red dashed lines indicate the 95% limits of agreement, and orange lines indicate the mean difference between the two methods. **(e)** Box-and-whisker plot of distances measured by US, Visual, and Tactile, indicating no significant difference between US vs. Visual (*p* = 0.2434) or US vs. Tactile (*p* = 0.9990). The distances measured by Tactile were significantly greater than the Visual (*p* = 0.0218). CEJ: cementoenamel junction. US: ultrasound.

Then, two examiners E1 and E2 (J.C. and H.C.) each individually measured distances between the lower border of the top ball bearing and the CEJ marked by the clinician with black and blue dots (Fig. 1a, left). The measurements were performed with a caliper under a dissecting microscope. The distances measured by E1 and E2 were averaged for comparison to ultrasound measurements. The bias between E1 and E2 was analyzed to evaluate the inter-examiner variation of the distance reading of clinical assessments.

### Detecting CEJ by ultrasound imaging

All teeth were fixed on custom-made 3D-printed holders (12 × 20 cm) by glue (Fig. S1) and immersed under water for coupling. The ultrasound imaging used a 40-MHz transducer (UHF57x, Visualsonics) and a commercially available ultrasound imaging system (Vevo F2, Visualsonics). All images have the same dimension of 14 mm width by 11 mm depth, with a focal depth at 7 mm. The frames with two steel ball bearings appearing simultaneously were analyzed on VevoLab software by a blinded examiner E0, an experienced ultrasound imaging researcher (B.Q.). The CEJ was identified by localizing the small V-shaped valley at the meeting point of enamel and cementum^17,28^. The distance between the center of the top ball bearing and the CEJ was measured and then subtracted the radius of the ball bearing (0.25 mm) for comparison to the distance measured by visual examination and tactile sensation (Fig. 1a, right).

The relative differences between ultrasound measurement and two clinical methods were calculated using Eqs. (1), (2):

1 $${\text{Relative~}}\;{\text{difference~}}\;{\text{between~}}\;{\text{ultrasound~}}\;{\text{and~}}\;{\text{visual}} = \left( {{\text{D}}_{{{\text{US}}}} - {\text{D}}_{{\text{V}}} } \right)/{\text{D}}_{{\text{V}}} ~\left( \% \right)$$

2 $${\text{Relative~difference~between~ultrasound~and~tactile}} = \left( {{\text{D}}_{{{\text{US}}}} - {\text{D}}_{{\text{T}}} } \right)/{\text{D}}_{{\text{T}}} ~\left( \% \right)$$

Where D_US_, D_V_, and D_T_ refers to the distance between the top ball bearing and the CEJ identified by ultrasound imaging, visual examination, and tactile sensation, respectively.

### Types and classifications of extracted teeth

Four teeth were excluded out of all 157 teeth because the ultrasound imaging did not capture the two ball bearings in the same frame. The included extracted teeth (*n* = 153) were divided by types into three groups: incisors (*n* = 42), cuspids (*n* = 20), and molars/premolars (*n* = 91).

The CEJ and cervical region were classified as described by Pini-Prato et al. and the AAP classification system^27,29^. Class A- included teeth (*n* = 75) with an intact CEJ that was visually detectable with or without a step affected by a shallow NCCL. Class B- (*n* = 78) included teeth with a visually undetectable CEJ affected by NCCL and demonstrated a cervical step of 1 mm or less. For the Class B- teeth, the mesial and distal CEJ as well as the color differences between the enamel and the dentin provided visual clues to the location of the CEJ.

We noted that the depths of the NCCLs can be measured accurately with ultrasound imaging. Therefore, we also classified teeth into two subgroups: cervical lesions shallow (CL-S) (*n* = 102) for shallow lesions equal to or less than 0.2 mm and cervical lesions deep CL-D (*n* = 51) for deep lesions greater than 0.2 mm.

### Statistical analysis

Statistical analysis was performed with GraphPad Prism 9 (San Diego, CA) to study the agreement between ultrasound and clinical measurements across all teeth and sub-groups. Bland-Altman analysis was performed to evaluate the bias and limits of agreement. A histogram indicated the frequency distribution of the relative differences. Paired and unpaired t-test and ANOVA with post hoc test determined differences among measurements. Pearson correlation was performed to calculate the correlation coefficient and significance. The significance level was determined as 0.05.

We conducted a statistical power analysis^30^ via Python to plot the power as a function of sample size and to calculate the minimum detectable mean difference for desired power of 80%. This analysis was performed using a two-tailed test with a 5% significance level (α = 0.05), and a minimum difference of 0.2 mm, 0.25 mm, or 0.3 mm.

## Results

We compared the agreement and variance of the CEJ localized by ultrasound and the clinical measurements (visual examination and tactile sensation) of all 153 teeth. The sample size of 153 had a power of 80% (sufficiently high by convention^31^) to detect a minimum difference of 0.25 mm (Fig. S2). We performed sub-group analysis across classifications of A- and B-, CL-S and CL-D, and three types of teeth. The descriptive statistics of the teeth used in this study based on sub-groups of classifications and tooth types are summarized in Table 1.

**Table 1:** Descriptive statistics.

	Classification		Classification		Tooth type			Overall (*n* = 153)
	A- (*n* = 75)	B- (*n* = 78)	CL-S (*n* = 102)	CL-D (*n* = 51)	Incisors (*n* = 42)	Cuspids (*n* = 20)	Molars/pre-molars (*n* = 91)	
Ultrasound (mm)	3.02 ± 0.67	3.23 ± 0.85	3.06 ± 0.74	3.26 ± 0.83	3.37 ± 0.73	3.48 ± 0.97	2.94 ± 0.69	3.13 ± 0.77
Visual (mm)	3.10 ± 0.61	3.00 ± 0.86	3.12 ± 0.71	2.90 ± 0.80	3.22 ± 0.82	3.31 ± 0.72	2.91 ± 0.69	3.05 ± 0.75
Tactile (mm)	3.14 ± 0.63	3.11 ± 0.94	3.19 ± 0.75	3.00 ± 0.88	3.27 ± 0.89	3.38 ± 0.77	3.00 ± 0.75	3.13 ± 0.80

### Identification of the CEJ via ultrasound images and comparison to clinical methods

Our first step was to use Bland-Altman analysis to compare the visually-identified distance with the ultrasound-identified distance (Fig. 1b). We found that 95% of the values fell within a range of 2.44 mm with a bias of 0.08 mm (ultrasound-based measurements are larger); the correlation analysis of the two distances showed a significant correlation with Pearson’s *r* = 0.6650 (Fig. S3a). Similarly, when ultrasound-based values were compared to tactile-based assessment, the 95% range was 2.66 mm with no systemic bias (Fig. 1c); the correlation analysis still showed a significant correlation but the Pearson’s r (0.6286) was 5.5% lower (Fig. S3b). The visual and tactile measurements showed a 95% CI of 1.42 mm with a bias of − 0.08 mm (Fig. 1d), and a Pearson’s r of 0.8924 (Fig. S3c). The ANOVA test indicated that the ultrasound measurements were not significantly different from either the visual or tactile measurements (*p* = 0.2434 and 0.9990, respectively), while the tactile measurements were significantly higher than the visual measurements (*p* = 0.0218) (Fig. 1e). To further quantify the error between the three different distances, we prepared a histogram (Fig. S3d), which showed that 71% (*n* = 108) had a relative difference within ± 20% for tactile versus ultrasound and 73% (*n* = 112) for visual versus ultrasound.

**Fig. 2:**
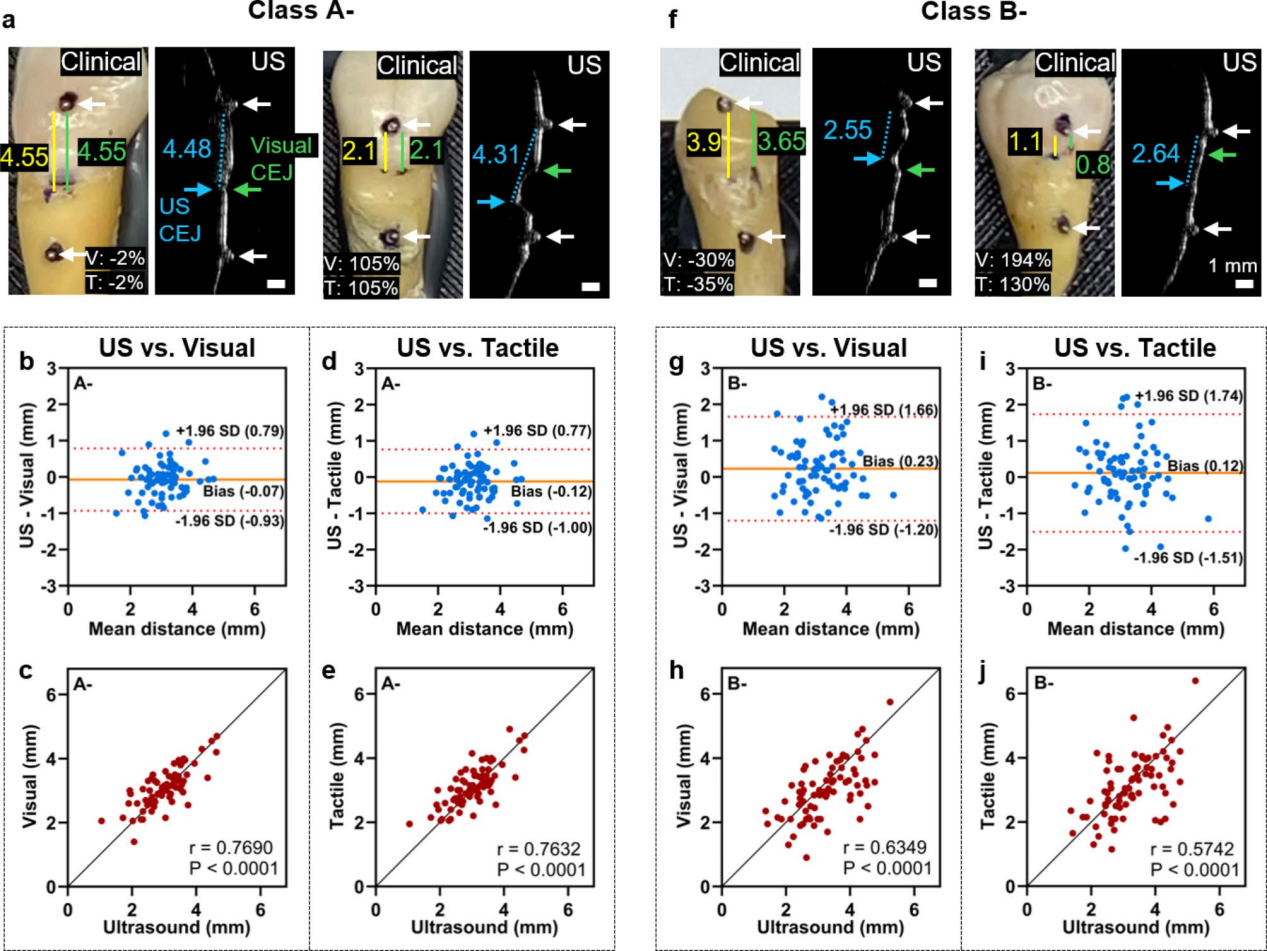
Comparisons of the CEJ identification between ultrasound and clinical measurements on Class A- (left) and Class B- (right). **(a)** and **(f)** show representative teeth in Class A- (visually detectable CEJ) and Class B- (visually undetectable CEJ), respectively. The agreement was characterized by Bland-Altman plot and Pearson correlation on teeth in **(b)–(e)** Class A- and **(g)–(j)** Class B-. Class A- shows higher agreement than Class B- for both US vs. Visual and US vs. Tactile.

### Impact of CEJ integrity on ultrasound measurement accuracy

We next studied how the CEJ integrity impacted the accuracy of ultrasound assessment. Subgroup analysis thus used the American Academy of Periodontology classifications (Class A- and Class B-)^27^.

For Class A- (Fig. 2a), comparing the distances measured by ultrasound versus visual, the Bland-Altman analysis presented a mean bias of − 0.07 mm with 95% limits of agreement from − 0.93 mm to 0.79 mm (Fig. 2b). The correlation coefficient was 0.7690 (*p* < 0.0001) (Fig. 2c). Comparing the distances measured by ultrasound versus tactile, the mean bias was − 0.12 mm and 95% limits of agreement was from − 1.00 mm to 0.77 mm (Fig. 2d). The correlation coefficient was 0.7632 (*p* < 0.0001) (Fig. 2e). For Class B- (Fig. 2f), the mean bias of ultrasound versus visual was 0.23 mm and the 95% limits of agreement was from − 1.20 mm to 1.66 mm (Fig. 2g). The correlation coefficient was 0.6349 (*p* < 0.0001) (Fig. 2h). The mean bias of ultrasound versus tactile was 0.12 mm and the 95% limits of agreement was from − 1.51 mm to 1.74 mm (Fig. 2i). The correlation coefficient was 0.5742 (*p* < 0.0001) (Fig. 2j). Comparing ultrasound versus visual, the 95% range of Class B- (2.86 mm) was 66% larger than that of Class A- (1.72 mm), and he correlation coefficient of Class A- was 21% higher than that of Class B-. Comparing ultrasound versus tactile, the 95% range of Class B- (3.25 mm) was 84% larger than that of Class A- (1.77 mm), and the correlation coefficient of Class A- was significantly higher than that of Class B-.

The ANOVA test indicated that the ultrasound measurements were not significantly different from either the visual or tactile measurements for Class A- (Fig. S4a). For Class B-, the ultrasound measurements were significantly higher (*p* < 0.05) than the visual measurements. There was no significant difference (*p* > 0.05) between ultrasound measurement and tactile sensation on B- teeth (Fig. S4b). On 80% of Class A- teeth (*n* = 60), the ultrasound and visual measurements showed a relative difference within ± 20%; while for Class B- the ratio was only 62% (*n* = 48) (Fig. S4c). 81% teeth in Class A- (*n* = 61) had a relative difference between ultrasound and tactile measurements within ± 20%—this was only 66% for Class B- (*n* = 51) (Fig. S4d).

Moreover, we rounded the visual and tactile measurements in accordance with the clinical standard^32,33^ for comparison (Fig. S5). In periodontal standard, the probing measurements were rounded to integers making it challenging to monitor small periodontal disease progression. For this reason, comparing rounded clinical measurements with the ultrasound imaging could be a problem as ultrasound imaging can reach sub-100 μm resolution.

The correlation between ultrasound and rounded visual examination decreased from 0.77 to 0.68 in Class A-. The 95% limits of agreement increased from [− 0.93, 0.79 mm] to [− 1.13, 0.96 mm], Similarly, the correlation decreased from 0.76 to 0.67 for tactile sensation in Class A-, and the 95% limits of agreement increased from [− 1.00, 0.77 mm] to [− 1.20, 0.96 mm]. Interestingly, minor changes were observed in Class B-. The correlation between rounded visual examination and ultrasound decreased from 0.63 to 0.61 in Class B-. the 95% limits of agreement increased from [− 1.20, 1.66 mm] to [− 1.35, 1.73 mm]. The correlation decreased from 0.57 to 0.53 for tactile sensation in Class B-, and the 95% limits of agreement increased from [− 1.51, 1.74 mm] to [− 1.65, 1.77 mm]. These results further confirmed that rounded clinical measurements lowered the agreement with ultrasound measurements and should not be taken as the gold standard in the future ultrasound-based periodontal diagnosing.

**Fig. 3:**
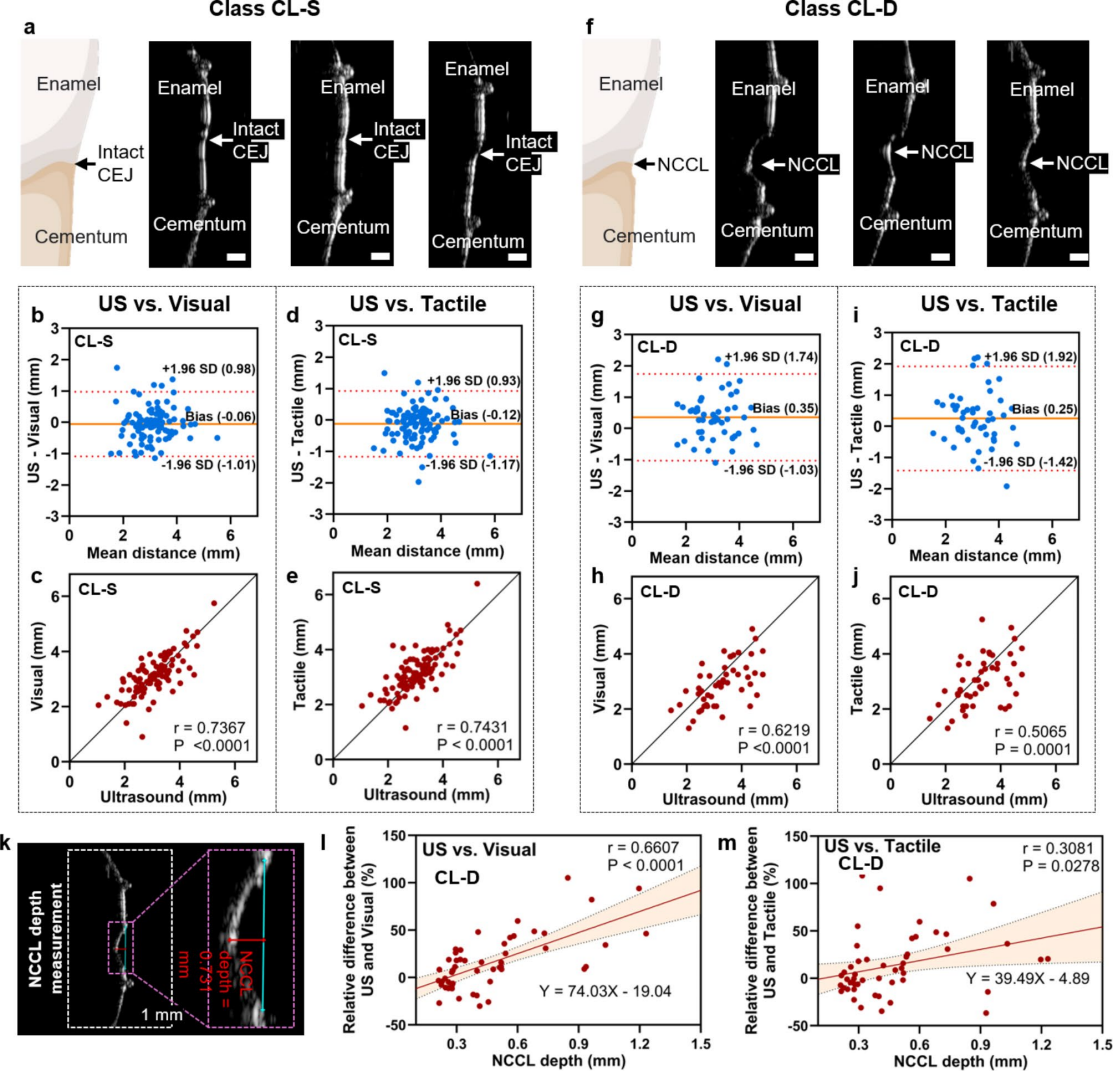
Comparison of CEJ identification between ultrasound and clinical measurements on Class CL-S (left) and Class CL-D (right). **(a)** and **(f)** show representative teeth in Class CL-S (shallow cervical lesions) and Class CL-D (deep cervical lesions), respectively. The agreement was characterized by Bland-Altman plot and Pearson correlation on teeth in **(b)–(e)** Class CL-S and **(g)–(j)** Class CL-D. Class CL-S shows higher agreement than Class CL-D for both US vs. Visual and US vs. Tactile. **(k)** Measurement of cervical lesion (i.e., the step) depth using ultrasound imaging. **(l)–(m)** The variance between US vs. Visual and US vs. Tactile showed a significant correlation with the depth of NCCL on teeth in Class CL-D, indicating that deeper cervical lesions can cause greater bias between ultrasound-based and clinical CEJ identification. Red line represents the regression line, and black dashed lines are boundaries of the 95% confidence interval.

### Impact of NCCL depth on ultrasound measurement accuracy

We noted a subgroup of Class B- teeth with deeper cervical lesions demonstrating more discrepancies among the detection methods. Therefore, we combined Class A- (all with no steps or shallow steps ≤ 0.2 mm) and 27 teeth of Class B- with shallow steps into CL-S (Fig. 3a), and assigned Class B- teeth with deep steps > 0.2 mm to CL-D (Fig. 3f).

Next, we analyzed the impact of cervical lesions on the CEJ identification accuracy using ultrasound by comparing the measurements in CL-S and CL-D. Comparing the distances measured by ultrasound versus visual examination, the Bland-Altman analysis presented a mean bias of – 0.06 mm with 95% limits of agreement from − 1.01 mm to 0.98 mm for CL-S (Fig. 3b). The correlation coefficient was 0.7367 (*p* < 0.0001) (Fig. 3c). For CL-D, the mean bias was 0.35 mm and the 95% limits of agreement was from − 1.03 mm to 1.74 mm (Fig. 3g). The correlation coefficient was 0.6219 (*p* < 0.0001) (Fig. 3h). To compare the distances measured by ultrasound versus tactile sensation, the mean bias was − 0.12 mm and 95% limits of agreement was from − 1.17  to 0.93 mm for CL-S (Fig. 3d). The correlation coefficient was 0.7431 (*p* < 0.0001) (Fig. 3e). For CL-D, the mean bias was 0.25 mm and the 95% limits of agreement was from − 1.42  to 1.92 mm (Fig. 3i). The correlation coefficient was 0.5065 (*p* < 0.0001) (Fig. 3j). The 95% range of CL-D (2.77 mm) was 39% larger than that of CL-S (1.99 mm). The correlation coefficient of Class CL-S was 18% higher than that of Class CL-D.

The ANOVA test indicated that the ultrasound measurements were not significantly different from either the visual or tactile measurements for Class CL-S (Fig. S6a). For Class CL-D, the ultrasound measurements were significantly higher than the visual measurements (*p* < 0.05), but ultrasound showed no significant difference than the tactile measurements (Fig. S6b). In Class CL-S, 76% teeth (*n* = 78) showed that the relative difference between ultrasound and visual measurements was within ± 20%; while for Class CL-D the ratio was only 59% (*n* = 30) (Fig. S6c). Here, 80% of teeth in CL-S (*n* = 82) had a relative difference between ultrasound and tactile measurements within ± 20%; for Class CL-D the ratio was only 59% (*n* = 30) (Fig. S6d).

The depth of the cervical lesion was measured as shown in Fig. 3k. For teeth in CL-D, the depth and the relative difference between ultrasound versus visual examination showed a significant positive correlation with Pearson’s *r* = 0.6607 (*P* < 0.0001), indicating that deeper cervical lesions caused greater bias across two methods (Fig. 3l). The depth of the cervical lesions and the relative difference between ultrasound versus tactile sensation also showed a significant positive correlation (*r* = 0.3081, *P* = 0.0278) (Fig. 3m), but the correlation coefficient was 53% lower than ultrasound versus visual examination.

**Fig. 4:**
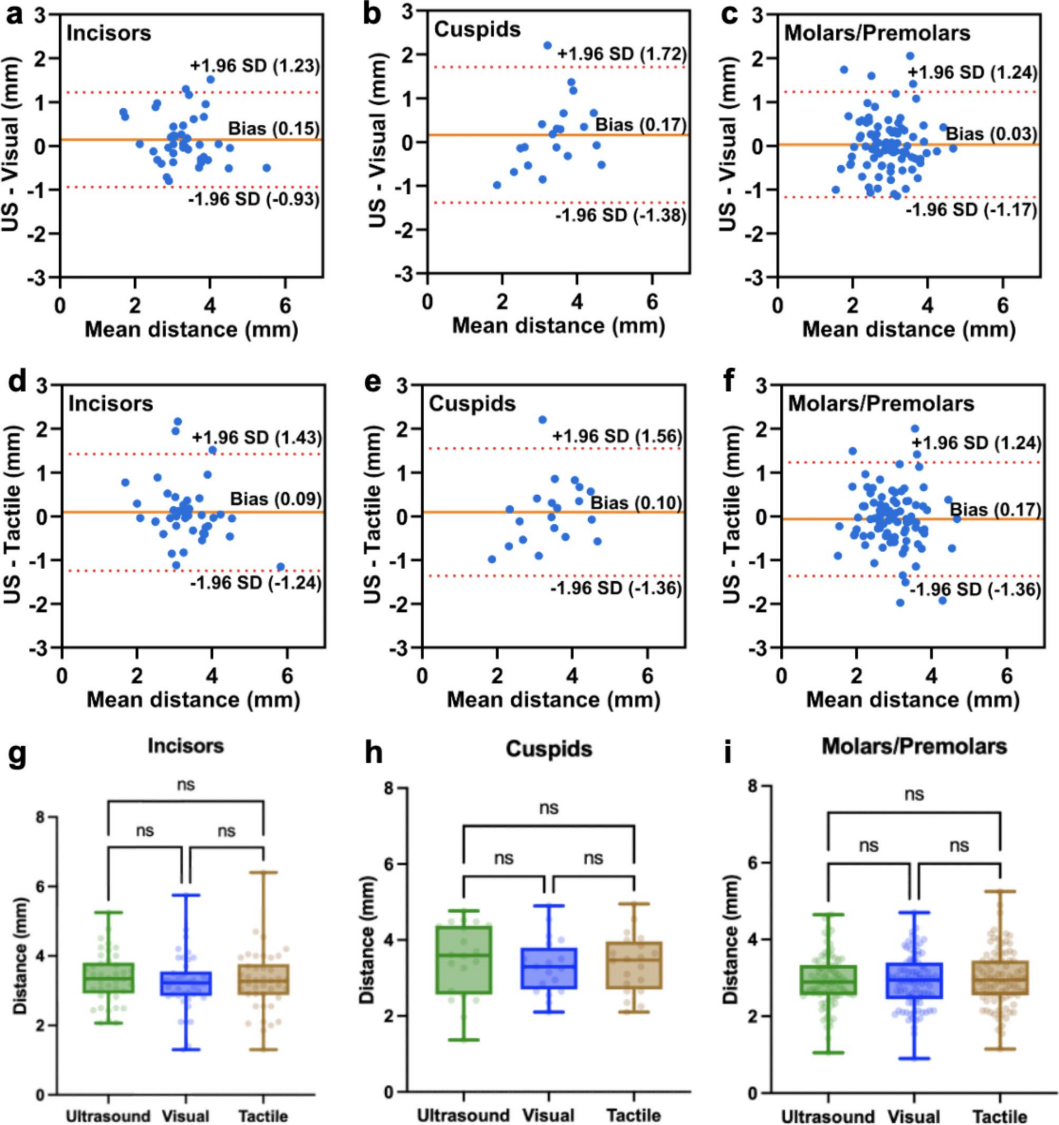
Comparisons of the CEJ identification between ultrasound and clinical methods on different tooth types. **(a)–(c)** and **(d)–(f)** show Bland-Altman plots of US vs. Visual and US vs. Tactile for incisors, cuspids, and molars/premolars, respectively. **(g)–(i)** Box-and-whisker plots showing no significant differences across ultrasound, visual, and tactile measurements for all three types of teeth (*p* > 0.05).

### Impact of tooth type on ultrasound measurement accuracy

We next investigated whether ultrasound analysis was impacted by tooth type: incisors, cuspids, or molars/premolars.

In the aspect of ultrasound versus visual measurements, the incisors showed a mean bias of 0.15 mm and a 95% range from − 0.93 to 1.23 mm (Fig. 4a). The correlation coefficient was 0.8356 (*p* < 0.0001) (Fig. S7a). For cuspids, the mean bias was 0.17 mm and the 95% range was from − 1.38  to 1.72 mm (Fig. 4b). The correlation coefficient was 0.5997 (*p* = 0.0052) (Fig. S7b). For molars/premolars, the mean bias was − 0.09 mm and the 95% range was from − 1.17  to 1.24 mm (Fig. 4c), and the correlation coefficient was 0.6891 (*p* < 0.0001) (Fig. S7c). The range of 95% confidence interval of cuspids was 44% and 29% larger than for incisors and molars/premolars, respectively, because of the limited number of cuspids available from the clinic^34–36^.

Then the ultrasound and tactile measurements were compared. The incisors had a mean bias of 0.09 mm and 95% range of − 1.24  to 1.43 mm (Fig. 4d), and a correlation coefficient of 0.8771 (*p* < 0.0001) (Fig. S5d). The cuspids presented a mean bias of − 0.10 mm and 95% range of – 1.36  to 1.56 mm (Fig. 4e), and a correlation coefficient of 0.6589 (*p* = 0.0016) (Fig. S7e). The molars/premolars showed a mean bias of − 0.17 mm and 95% range of − 1.36  to 1.24 mm (Fig. 4f), and a correlation coefficient of 0.6616 (*p* < 0.0001) (Fig. S7f).

Distances measured using ultrasound, visual, and tactile measurements showed no significant difference for all three types of teeth (Fig. 4g–i). 59% incisors (20 out of 34), 56% cuspids (5 out of 9), and 77% molars/premolars (53 out of 69) had a relative difference between ultrasound and visual measurement within ± 20% (Fig. S8a). 65% incisors (*n* = 22), 67% cuspids (*n* = 6), and 78% molars/premolars (*n* = 54) had a relative difference between ultrasound and tactile measurement within ± 20% (Fig. S8b).

### Inter-rater reliability of clinical probing reading

Next, the inter-rater reliability was studied by comparing the reading of visual and tactile measurements between E1 and E2.

The visual distances measured by E1 was higher than E2 with a mean bias of 0.18 mm. The 95% range was 1.30 mm, with limits of agreement from − 0.47 mm to 0.83 mm (Fig. S9a). The visual measurements by E1 were significantly higher than E1 (*p* < 0.05, paired t-test) (Fig. S9b). The ICC was 0.86.

Similarly, the tactile distances measured by E1 was higher than E2 with a mean bias of 0.17 mm. The 95% range was 1.18 mm, with limits of agreement from − 0.42 mm to 0.76 mm (Fig. S9c). The tactile measurements by E1 were significantly higher than E1 (*p* < 0.05, paired t-test) (Fig. S9d). The ICC was 0.89.

## Discussion

Previously, Nguyen et al.^16^ have studied the ultrasound CEJ identification on six porcine central incisors by comparing to micro-computed tomography. The 95% limits of agreement were reported as − 0.49 to 0.17 mm. In this study, we evaluated the accuracy of ultrasound CEJ identification by comparing to clinical assessments on 153 human extracted teeth for the first time.

Overall, the mean bias of CEJ localized by ultrasound imaging compared to clinical visual and tactile sensing were both < 0.1 mm, which is less than the mean bias of inter-rater distance measurement on the same labelling (0.18 mm for visual; 0.17 mm for tactile). For teeth in Class A- and teeth in Class CL-S, there was no significant difference between ultrasound and visual or ultrasound and tactile.

In Class A-, the feature of CEJ (small V-shaped valley) is clear on the ultrasound imaging. All teeth in Class A- showed either no cervical lesions or lesions with a depth no greater than 0.2 mm. The distances from the reference point to the CEJ identified by ultrasound showed no significant difference compared to either visual examination or tactile sensation. The agreement of ultrasound versus visual and tactile measurements are high with 95% limits of agreement less than ± 1 mm, which was within the 1-mm precision of the clinical rounded measurement for CEJ identification^32,33^. The error source of group A- comes from teeth with no obvious CEJ features or more than one possible indentation.

In Class B-, the distances measured by ultrasound were significantly higher than the visual measurements (*p* < 0.05), and the bias showed a significant positive correlation with the cervical lesion depth. This is because the CEJ on teeth in Class B- was difficult to detect visually. Also, 64% teeth in Class B- showed a NCCL depth greater than 0.2 mm.

In cases of deep cervical lesions that affected CEJ (CL-D), the CEJ (visual) was determined by drawing a line from the intact CEJ at the mesial and the distal aspect of the teeth. The clinical-ultrasound correlation for CL-D was weaker compared to CL-S, and the discrepancy between clinical and ultrasound measurements showed a positive correlation with lesion depth. The appearance, texture, and shape of tooth surface in the CEJ area was destructed by NCCL, thus it causes difficulty for CEJ identification for both clinical and ultrasound measurements.

The errors between ultrasound and clinical measurements showed no significant difference across tooth types. However, the numbers of incisors, cuspids, and molars/premolars were different based on different extraction rates and clinical availability. The smaller number of cuspids resulted in a larger confidence interval. In the future, having a similar number in each group of different tooth types will provide more consistent statistical power for confidence interval comparison.

The accuracy of clinical probing CEJ identification highly depends on the clinician’s experience^37^. The inter-rater reliability showed that even measuring the same distance labeled by the same clinician, the reading by different examiners can be significantly different. In this study, the visual and tactile distances were measured with a caliper under a microscope and the precision was 0.1 mm. However, clinically, the precision of the traditional periodontal probing for CEJ identification was rounded as 1 mm^32,33^, which further limits the accuracy of landmarks localization.

Ultrasound, instead, is an imaging-based method that present landmarks and their relative location with sub-100-µm resolution^38^, and the distance reading precision is pixel-level. The periodontal ultrasound imaging with intraoral transducers has shown excellent repeatability with ICC score as high as 0.917^39^.

The root causes of the bias between ultrasound and clinical measurements include several factors.

First, the CEJ identification by ultrasound was based on localizing V-shaped valley feature. It is examiner dependent and requires experience on ultrasound. Although atomic force microscopy (AFM) has demonstrated that enamel is slightly smoother than cementum (with average surface height deviations of 0.46 μm for enamel and 0.65 μm for cementum)^40^, ultrasound cannot distinguish such small differences because its resolution is limited by the point spread function, which is approximately 40 μm for axial resolution, and 90 μm for lateral resolution. Elevational resolution (slice thickness) depends on the acoustic lens of the transducer used in this study and was not characterized here but other studies have shown that it can be up to three fold higher than the in-plane resolution^41^. The density of normal enamel and cementum is 2820–3095 and 1240–1340 mg/cc, respectively^42^. Ultrasound entropy imaging has been proposed to quantitatively differentiate bones with different densities and micro-structures^43,43^. It could be a potential solution to identify the CEJ quantitatively by ultrasound and eliminate examiner bias.

Second, although widely applied in clinics, both the visual examination and tactile sensation suffer from operator dependency and limited by probing precision. To solve this problem, the CEJ identification by ultrasound need to be compared with histology, radiograph, or optical coherence tomography as the gold standard reference. The gold standard methods can also verify if the tooth has a CEJ classification of overlap, gap, or edge-to-edge^44^, which was not included in this study due to limits of reaching this information.

Third, only one examiner was involved to identify CEJ on ultrasound images. In the future, more examiners and replica measurements can be included to investigate the inter- and intra-rater reliability. Furthermore, these teeth all had a variety of diseases leading to their extraction during routine care; thus, we must be careful about extending conclusions to teeth still encapsulated in alveolar bone and gingival tissue. Fourth, the teeth had been stored for several months and routine aging may have altered their acoustic properties. Fifth, the presence of a filling at the original CEJ region should be considered in future studies, as we would be identifying the clinical restoration margin rather than the CEJ. Depending on the acoustic impedance of the restoration material, the filling may present stronger or weaker signals in ultrasound imaging. Finally, the CEJ is much more difficult to locate in acoustic images because of the similarity in acoustic impedance between cementum and enamel. Landmarks like the alveolar bone crest and gingival margin are much more obvious. Additional studies are underway to better understanding CEJ imaging in vivo in healthy and diseased human subjects.

## Conclusion

We investigated the agreement of ex vivo CEJ identification using ultrasound imaging and two clinical methods—visual examination and tactile sensing—in 153 extracted human teeth. For most (> 75%) teeth in Class A- and CL-S, the difference between ultrasound imaging and clinical methods was within ± 20%. Ultrasound can image the morphology of a tooth with a sub-100-µm resolution, thus provide more accurate information than the clinical probing with a 1-mm precision. We anticipate that advances of ultrasound imaging and its good agreement with clinical methods will further facilitate the clinical translation. Next steps for practical applicability include (1) performing in vivo CEJ identification in an expanded cohort including healthy and diseased subjects; (2) comparing ultrasound-based identification with gold standard references (e.g., radiography); (3) automatically localizing the CEJ by differentiating ultrasound signals from enamel and cementum due to their density and texture differences.

## Electronic supplementary material

Below is the link to the electronic supplementary material.

Supplementary Material 1

## Data Availability

The datasets generated during and/or analyzed during the current study are available from the corresponding author on reasonable request.
