# Supplementary material for: Ultrasound identification of the cementoenamel junction and clinical correlation through ex vivo analysis
Source: Sci Rep. 2024 Nov 13;14:27821. doi: 10.1038/s41598-024-79081-z (PMC11561095; doi:10.1038/s41598-024-79081-z)
Supplement: Supplementary file 1 — Supplementary Material 1 [file 41598_2024_79081_MOESM1_ESM.docx]

**SUPPLEMENTARY INFORMATION**

**Ultrasound Identification of the Cementoenamel Junction and Clinical Correlation through Ex Vivo Analysis**

Baiyan Qi^1#^, Lei Fu^1#^, Tamer Abdelrehim^1^, Jason J. Chang^2^, Harrison Chang^2^, Casey Chen^2^, Jesse V. Jokerst^1,3,4,*^

1 Aiiso Yufeng Li Family Department of Chemical and Nano Engineering, University of California, San Diego, La Jolla, CA 92093, USA

2 Herman Ostrow School of Dentistry, University of Southern California, 925 West 34th Street, Los Angeles, CA, USA

3 Material Science and Engineering Program, University of California, San Diego, La Jolla, CA 92093, USA

4 Radiology Department, University of California-San Diego, La Jolla, CA 92093, USA

**Corresponding author:** Jesse V. Jokerst

**Email:** [jjokerst@ucsd.edu](mailto:jjokerst@ucsd.edu)

^#^These authors contributed equally to this work


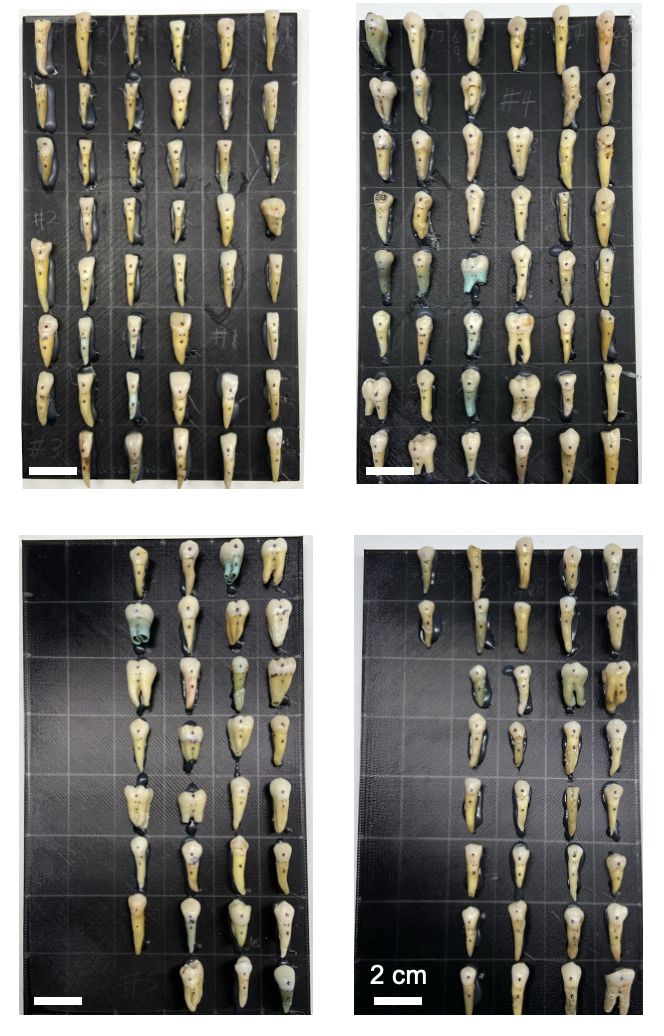


**Figure S1. All extracted teeth affixed to 3D printed holders.**


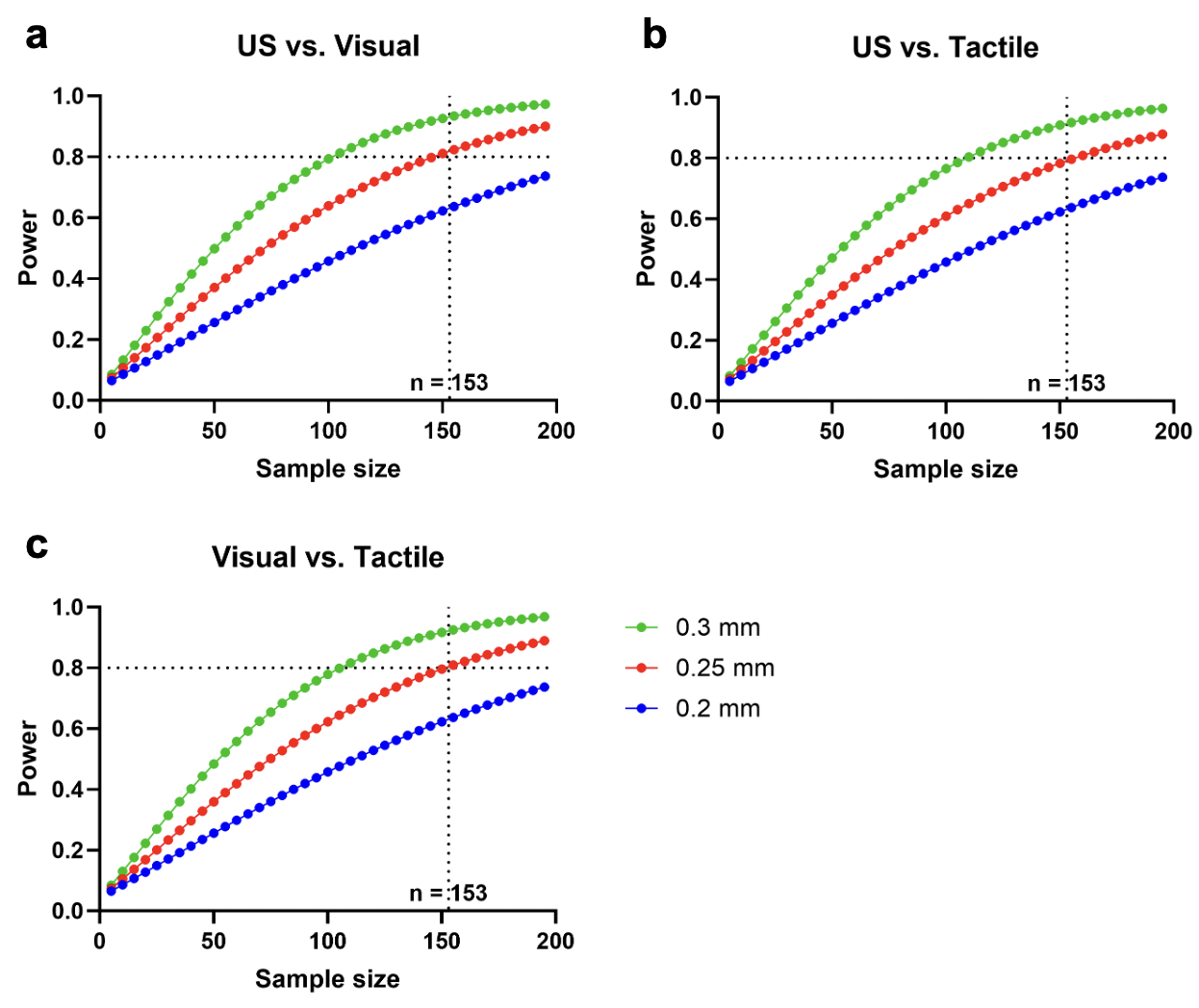


**Figure S2. Statistical power as a function of sample size using a two-tailed significance test (α = 0.05).** Mean differences were 0.2 mm (blue), 0.25 mm (red), and 0.3 mm (green). Effect size was calculated using standard deviations of **(a)** US vs. Visual, **(b)** US vs. Tactile, and **(c)** Visual vs. Tactile, respectively.


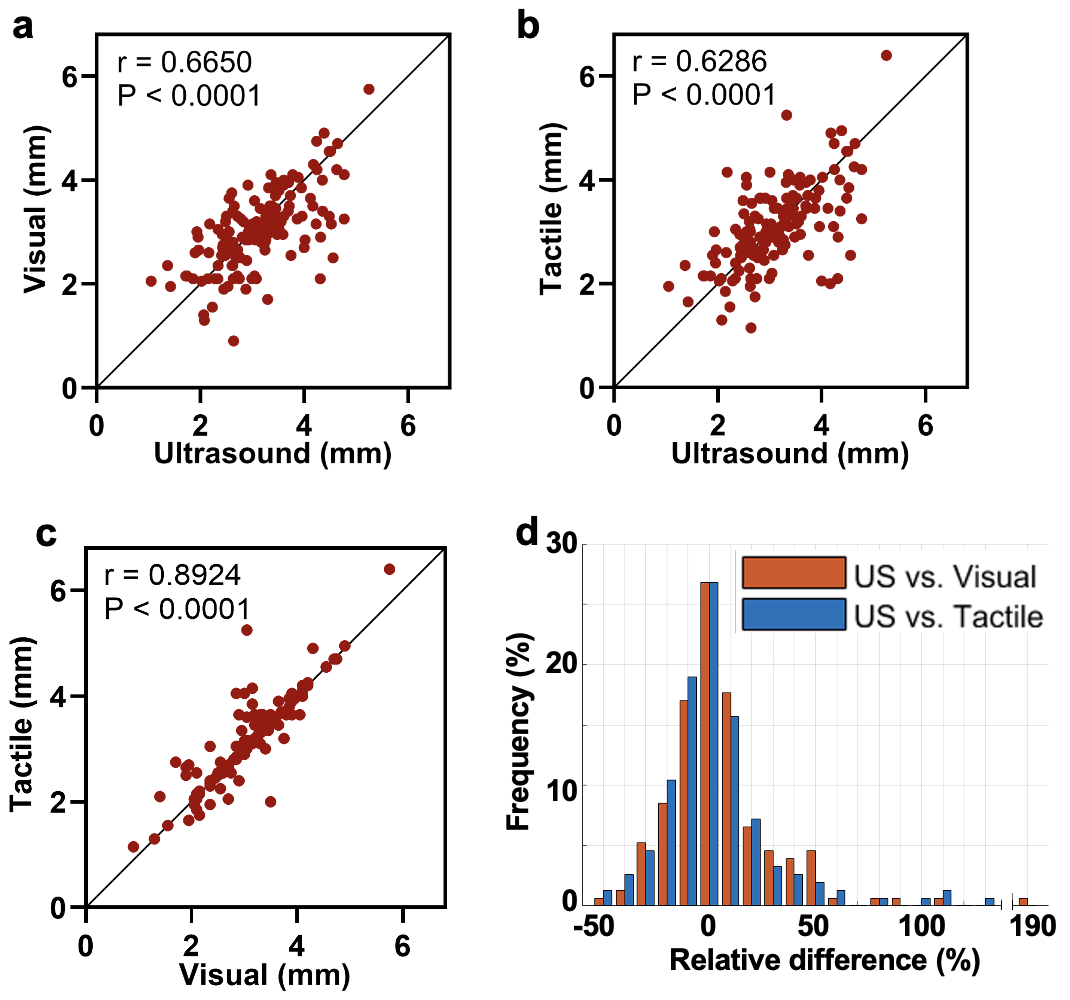


**Figure S3. Correlation and relative difference between ultrasound and clinical measurements of all teeth.** Pearson correlation plots of **(a)** US vs. Visual, **(b)** US vs. Tactile, and **(c)** Visual vs. Tactile. Black lines indicate the line of identity. **(d)** Frequency distributions of relative differences between US vs. Visual and US vs. Tactile. For 71% and 73% of the extracted teeth, the relative difference between US vs. Visual and US vs. Tactile was within ±20%, respectively.


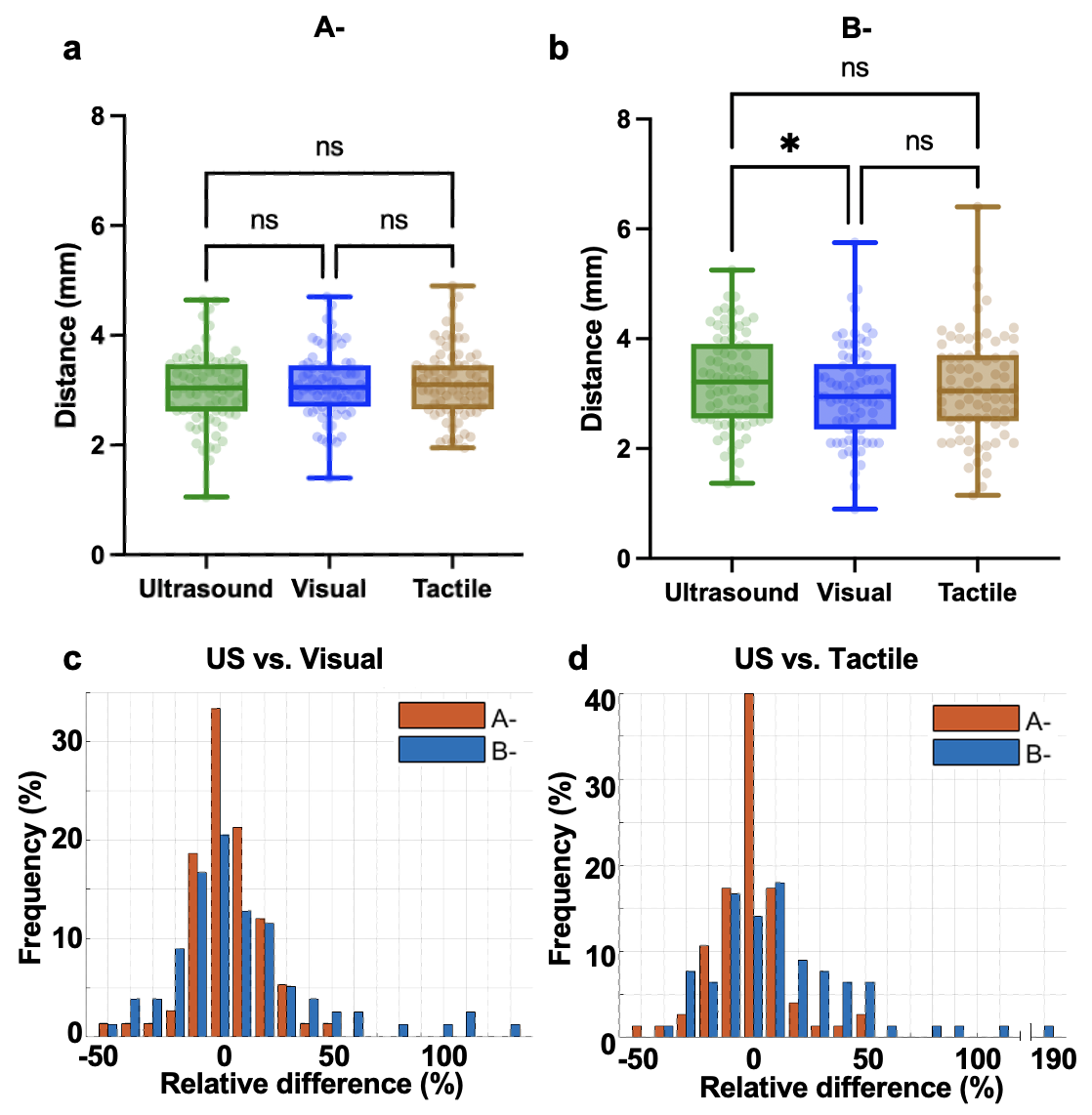


**Figure S4. Comparisons of the CEJ identification between ultrasound and clinical measurements on Class A- and Class B-. (a)-(b)** Box-and-whisker plots showing no significant differences across ultrasound, visual, and tactile measurements for Class A- (p > 0.05), while for Class B-, the ultrasound measurement were significantly higher than the visual examination (p < 0.05). **(c)** For 80% of Class A- and 62% of Class B-, the relative difference between US vs. Visual was within ±20%. **(d)** For 81% of Class A- and 66% of Class B-, the relative difference between US vs. Tactile was within ±20%.


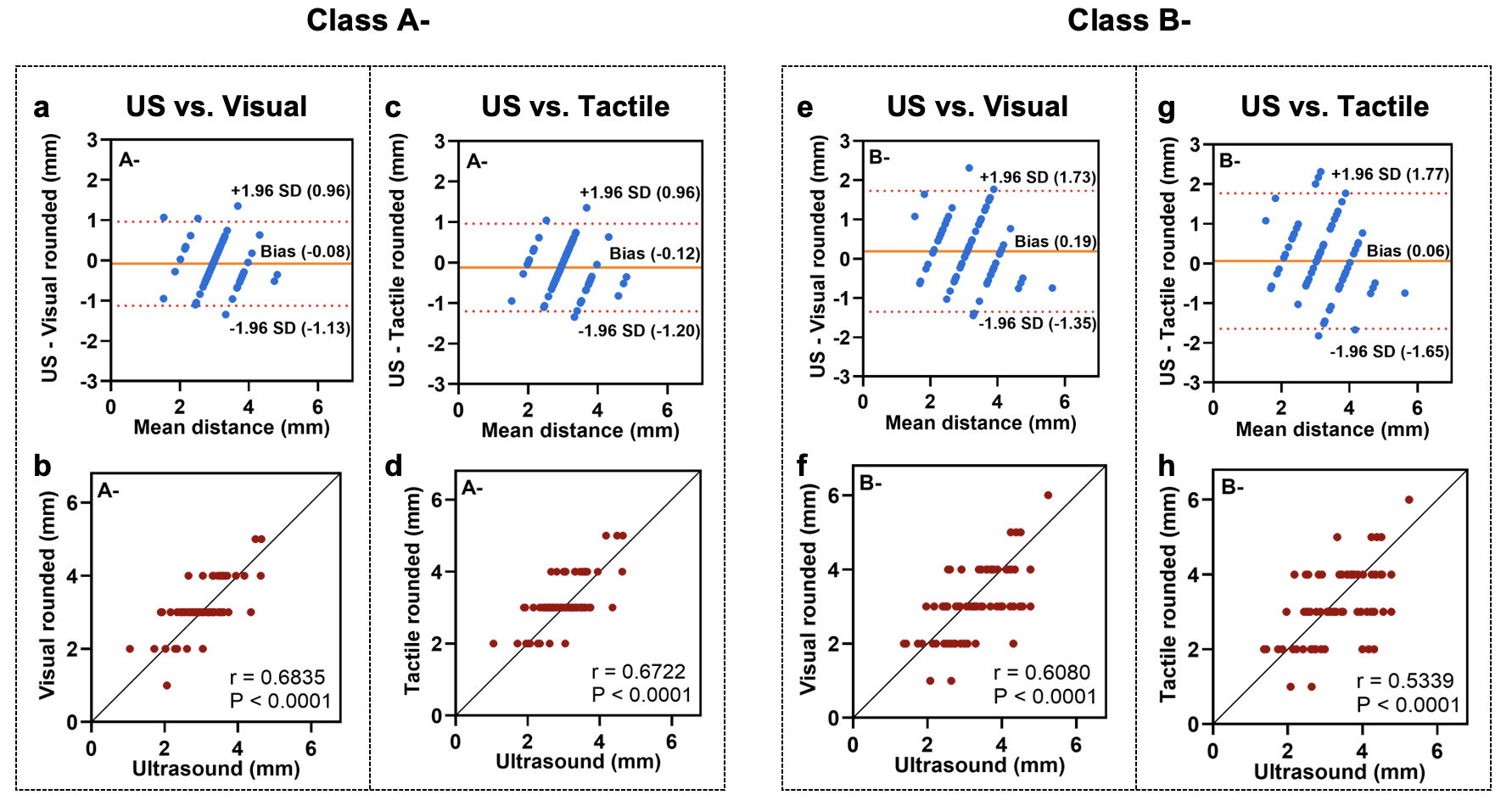


**Figure S5. Comparison of CEJ identification using ultrasound and rounded clinical measurements on Class A- and Class B-.** Bland-Altman plots and Pearson correlation plots of US vs. rounded Visual and US vs. rounded Tactile for teeth in Class A- and Class B-.


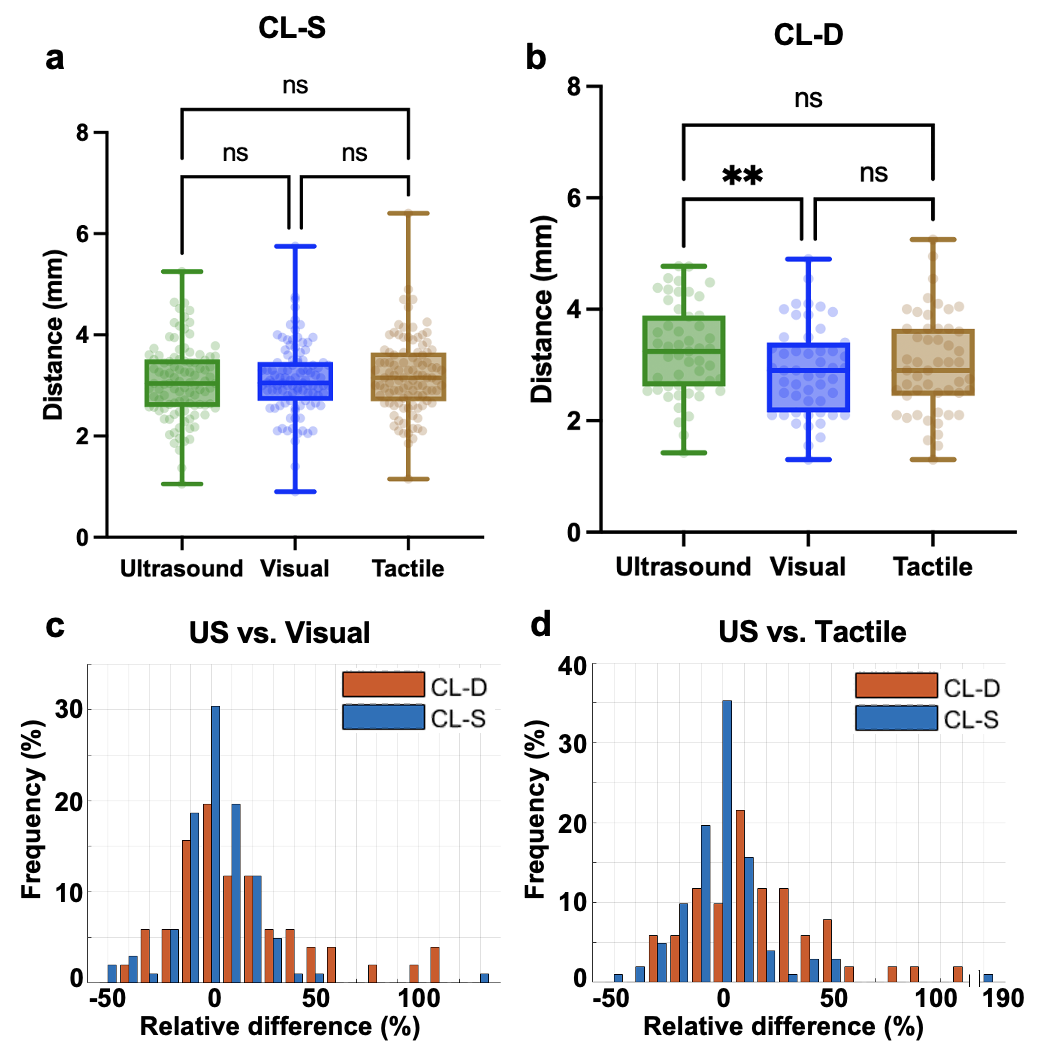


**Figure S6. Comparisons of the CEJ identification between ultrasound and clinical measurements on Class CL-S and Class CL-D. (a)-(b)** Box-and-whisker plots showing no significant differences across ultrasound, visual, and tactile measurements for Class CL-S (p > 0.05), while for Class CL-D, the ultrasound measurement were significantly higher than the visual examination (p < 0.05). **(c)** For 76% of Class CL-S and 59% of Class CL-D, the relative difference between US vs. Visual was within ±20%. **(d)** For 80% of Class CL-S and 59% of Class CL-D, the relative difference between US vs. Tactile was within ±20%.


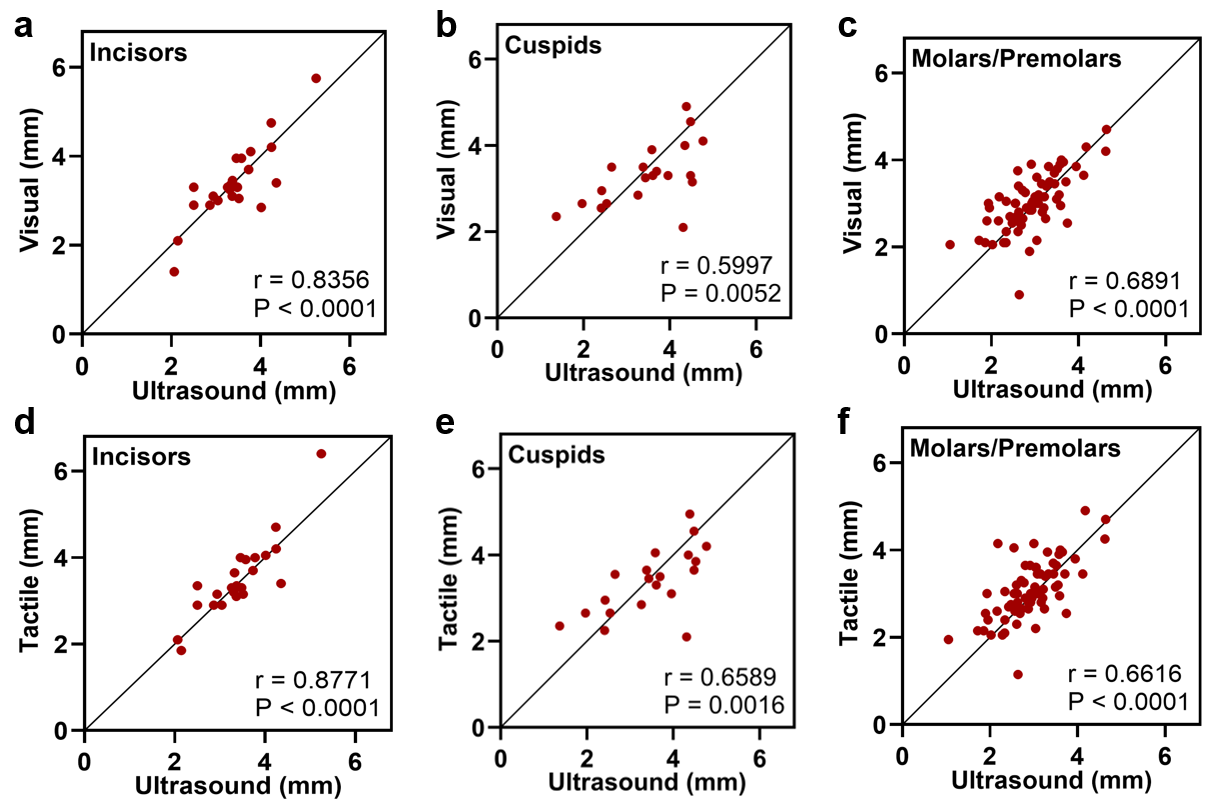


**Figure S7. Correlation between ultrasound and clinical measurements for incisors, cuspids, and molars/premolars.** **(a)-(c)** Pearson correlation plots of US vs. Visual for incisors, cuspids, and molars/premolars, respectively. **(d)-(f)** Pearson correlation plots of US vs. Tactile for incisors, cuspids, and molars/premolars, respectively.


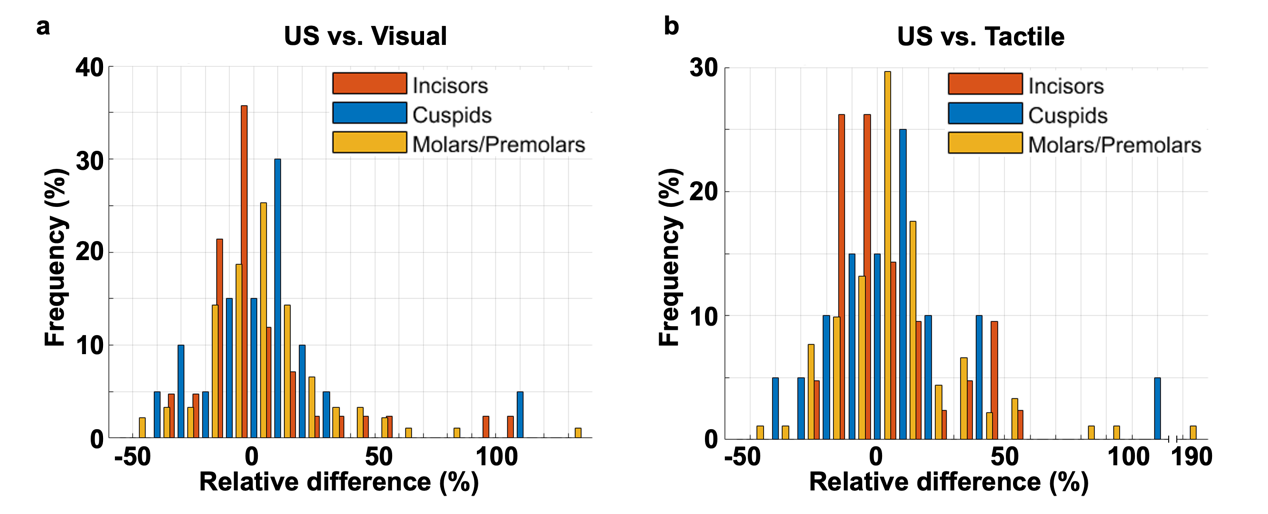


**Figure S8. Frequency distributions of relative differences between ultrasound and clinical measurements for incisors, cuspids, and molars/premolars. (a)** For 59% of incisors, 56% of cuspids, and 77% of molars/premolars, the relative difference between US vs. Visual was within ±20%. **(b)** For 65% of incisors, 67% of cuspids, and 78% of molars/premolars, the relative difference between US vs. Tactile was within ±20%.

**
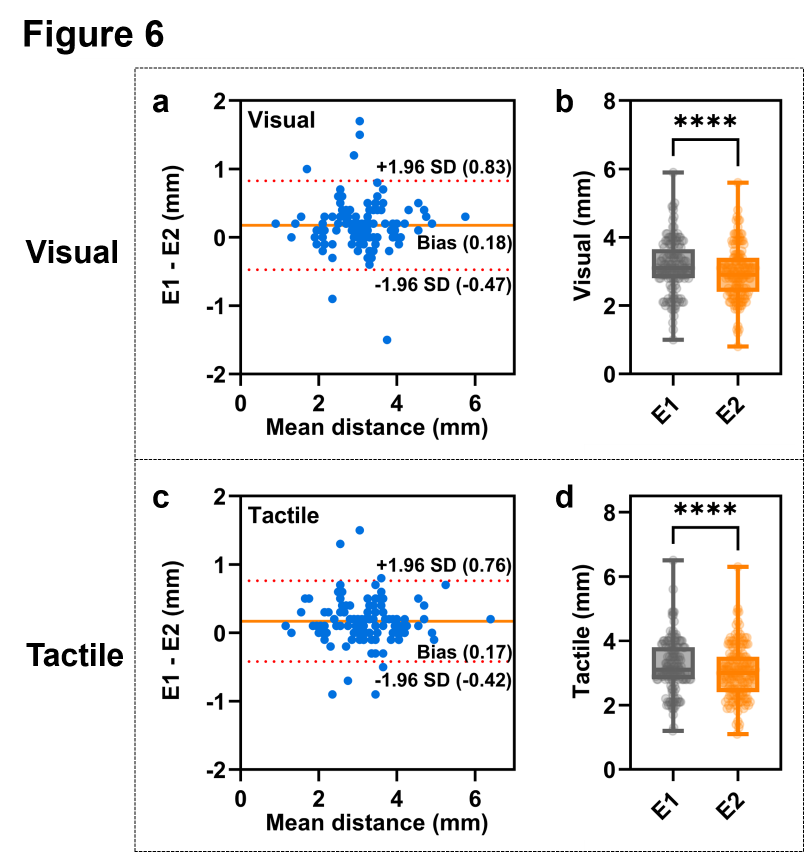
**

**Figure. S9 Characterization of inter-rater bias for clinical probing measurement.** The agreement of distances measured by E1 and E2 was characterized by Bland-Altman plots and box-and-whisker plots for **(a)-(b)** visual examination, and **(c)-(d)** tactile sensation, respectively. The distances read by E1 are significantly higher compared to E2 (p < 0.05, paired t-test) for both visual and tactile measurements.
